# Validation and stability analysis of guanine deaminase assay kit

**DOI:** 10.1016/j.heliyon.2024.e36210

**Published:** 2024-08-13

**Authors:** Qiang Sun, Haidi Gao, Yong Liu, Liqiang Wang, Jing Huang

**Affiliations:** a Department of Laboratory Medicine, The First Hospital of Jilin University, Changchun, Jilin Province, PR China; b Genetic Diagnosis Center, The First Hospital of Jilin University, Changchun, Jilin Province, PR China

**Keywords:** Guanine deaminase, Composite stabilizer, Xanthine oxidase, Uric acid oxidase

## Abstract

Guanine deaminase (GD)plays important roles in the diagnosis of liver function. However, there is no totally rapid and simple for the eatimation of GD activity in clinical application. Herein, we have constructed an enzymatic assay system with highly sensitive and strong stability for quantification of GD activity by highly double enzyme-coupling (xanthine oxidase and uric acid oxidase) and adding compound stabilizer in GD kit. In this study, we validated parameters, including reagent blank, sensitivity, accuracy, inter-batch difference, intra-batch difference, linear range. Furthermore, composite stabilizers, containing gentamicin sulfate, bovine serum albumin, and mannitol, were selected to improve stability of GD kit during long-term storage. The experimental results showed that the absorbance of the reagent blank was <0.2, the mean recovery rate was 103 %, the inter-batch and intra-batch diffeerence were <15 %, The linearity range was 0 U/L-50 U/L (R^2^ > 0.99). All indicators met the kit requirements for clinical applications. When gentamicin sulfate, bovine serum albumin, and mannitol were used as a stabilizer, the kit remained stable for 12 months without significant loss of enzymatic activity. These results indicated that GD kit possesses high sensitivity and strong stability, which can be used for routine biochemical applications and is of great significance for the diagnosis and differential diagnosis of liver diseases.

## 1 Introduction

Guanine deaminase (GD) is a metabolic enzyme mainly found in the liver, but rarely kidney and brain, which catalyzes the deamination of guanine to xanthine. Alanine aminotransferase and aspartate aminotransferase are indicators of liver function, hemolysis and myocardial injury can increase its contents, causing inaccurate results. GD is particularly abundant in liver cells, but itis almost absent in cardiomyocyte, leukocytes and erythrocytes. Hence, when liver cells are injured or diseased, GD is released, enhancing its activity in the blood. The effects caused by hemolysis and myocardial damage can be avoided. At present, there are no accurate and effective methods for diagnosing and treating, respectively, of liver diseases caused by damage to the hepatocytes. For instance, guanine deaminase is elevated in the early liver injury, while transaminase is normal. Therefore, GD activity is more valuable in monitoring the progression of liver disorders or other related diseases [[1], [2], [3]].

**Fig. 1:**
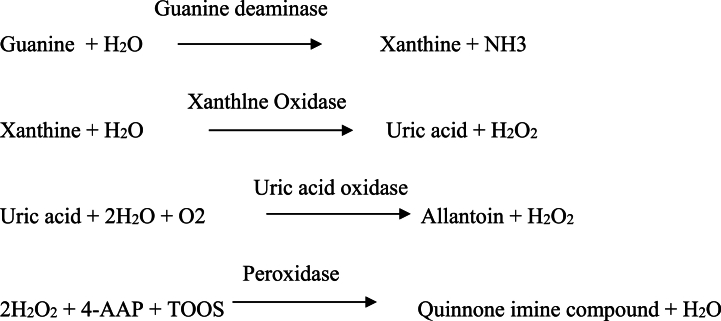
Enzyme catalysis reaction in GD kit.

Until now, various methods for measuring GD activity have been reported [[4], [5], [6], [7]]. The first approach indirectly determines the GD activity based on ammonia content; The second is the direct determination of xanthine production; The third measure uric acid generated during the hydrolysis of xanthine; The fourth determines the content of hydrogen peroxide; The fifth is the ^14^C labeling method, which uses ^14^C labeled guanine to generate xanthine, measuring the radioactive using a scintillation counter. However, due to the small extinction coefficient and low sensitivity in the first three methods, as well as the need for specialized equipment for the ^14^C labeling method, furthermore, these methods are not compatible with the automatic biochemical analyzer. Hence, these methods are not practical in routine clinical applications. In addition, in the conventional enzyme coupling reaction, GD in the serum converts guanine to xanthine in the first step; then the produced xanthine is catalyzed by xanthine oxidase (XOD) to produce uric acid and H_2_O_2_, which generates chromogenic reaction with an appropriate chromogen. However, the content of GD is particularly low in the blood, causing less H_2_O_2_ generation. Therefore, this method has markedly low sensitivity. In order to improve the sensitivity of this method, we developed a more accurate and sensitive method for measuring the GD activity in the blood. Based on the traditional methods, we utilizes the produced uric acid to more generate H_2_O_2_, improving the sensitivity of this method. Finally, H_2_O_2_ undergoes chromogenic reaction under the 4-aminoantipyrine (4-AA) and N-ethyl-N-(2-hydroxy-3-sulfopropyl)-3-methylaniline (TOOS), generating changes in absorbance. The absorbance change value is directly proportional to GD activity. The changed absorbance can be monitored using a biochemical analyzer. The working principle of GD kit is shown in Fig. 1. Therefore, the key enzymes component, which are the activity of XOD and uric acid oxidase directly determine the accuracy and sensitivity of GD kit.

GD activity detection is the current standard method for the diagnosis and assessment of liver disease, such as nonalcoholic fatty liver disease, alcoholic liver disease, hepatitis B virus infection, drug-induced hepatotoxicity, and metabolic liver disease, which is an accurate and specific indicator for the early diagnosis and prognosis of liver damage [8]. However, the practical application of using XOD faces challenges, XOD activity may loss during long-term storage time due to degradation or denaturation [9].Long-term storage stability is of importance for commercial kit application and use. Freeze drying has been widely used to maintain satisfactory biological activity of proteins during long-term storage [10]. Nevertheless, during freeze drying, proteins are affected by various factors, such as pH change, ice formation, crystal formation, and phase separation, leading to conformation significant changes and bioactivity loss [11]. Adding protectants is the most commonly used method to enhance biological activity during the long-term storage of proteins [12,13]. It is widely known that many stabilizers, such as sugars, polyols, amino acids, certain salts, and some proteins or poly-mers, have been extensively applied in storage [[14], [15], [16]]. Therefore, it is necessary to develop a more effective and stable method that remains XOD activity for a long time. Herein, we stabilize the XOD activity by adding a composite stabilizer composed of many macromolecular substances, including gentamicin sulfate, bovine serum albumin, and mannitol, to improve the stability of the GD kit.

**Fig. 2:**
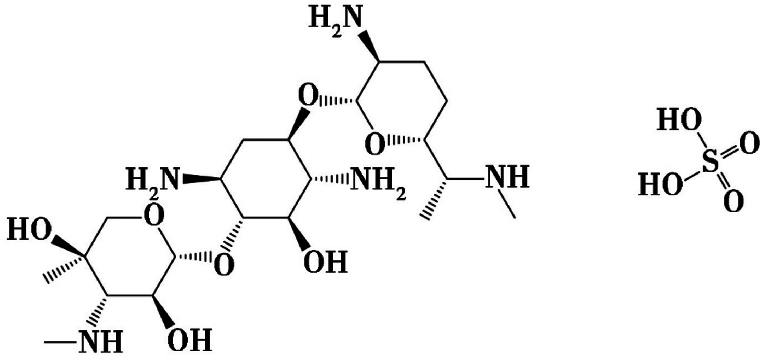
The chemical structure of the gentamicin sulfate.

Gentamicin sulfate (C_60_H_125_O_22_N_15_·H_2_SO_4_)(shown in Fig. 2)are polyhydroxy compounds, which can support and maintain enzymatic activity under heat, cold and dehydration, which was the main component of the composite stabilizer through forming hydrogen bonds to stabilize XOD activity [17,18]. This interaction can prevent changes in the spatial conformation of XOD, maintaining XOD activity for a long-term storage. The nature of the co-solvent plays a crucial role in the enzyme stabilizing process because it influences the ionic strength and the chemical affinity to certain molecular groups on the protein surface [19]. We select bovine serum albumin and mannitol to together prevent denaturation, stabilize and protect the stereoconformation of the XOD.

This study intended to develop GD kit with high sensitivity and strong stability. As far as we know, it is the first time to quantify GD activity in the serum applying this principle and composite stabilizer, which laid the foundation for the development of a new GD kit with potential clinical applications.

## 2 Materials and methods

### 2.1 Apparatus and materials

Hitachi automatic biochemical analyzer (7600-010), PH meter (sartorius); xanthine oxidase、peroxidase (POD)、uricase and catalase were obtained from TOYOBO CO. Tris hydrochloride and TOOS were purchased from TCI (Japan). Guanine deaminase was purchased from Sigma (USA), 4-AA was purchased from Beijing bailingwei Technology Co. Bovine serum albumin, gentamicin, mannitol were purchased from Merck. All chemicals are analytical grade unless otherwise indicated.

### 2.2 Extraction of GD

GD was extracted and purified as described by *Ali S* et al. from rabbit liver [20]. For this experiment, 100 g liver were rinsed thoroughly and repeatedly with isotonic 0.9 % (w/v) saline using a 5 mL syringe until they were completely white and cut into small pieces before grinding. First, liver homogenate with ultrasonic crushing were prepared with 2 L of saline under 4 °C condition. Next, the homogenates were centrifuged at 20000 rpm for 60 min, the supernatant were collected in a new tube. The erythrocytes in the supernatant were lysed using 5 % (mass fraction) sucrose solution. Finally, the lysate were centrifuged at 20000 rpm for 30 min, and the collected supernatant, which contained the GD activity, were stored at −20 °C until further use. The preparation was freshly diluted 15–20 fold with human serum for use in each assay.

### 2.3 Reaction system

The absorbance change values at 600 nm was used to quantify the reaction rate and characterize GD activity. The GD enzyme activity reaction system was described as follow. Briefly, the reaction system consisted of solution I and solution II. The solution I comprised 0.1 M Guanine hydrochloride, 40 U/L uricase, 17 KU/L catalase, 0.05 mM 4-AA and 35 mM Tris/HCI (pH 7.6). The solution II comprised 240 U/L XOD, 8 KU/L POD, 24 KU/L superoxide dismutase (SOD), 0.1 M TOOS and Tris/HCI (pH 6.5). The reaction started at 25 μL sample and 200 μL solution I, after 5 min was incubated with 200 μL solution II in another 5 min. The absorbance of the reaction system was measured at 600 nm using Hitachi automatic biochemical analyzer (7600-010) over 10 min in a total volume of 425 μL. The ratio of solution I to solution II is 4:1. Enzymatic activity was defined as the amount required catalyze guanine to produce 1 μmol of xanthine per minute under standard conditions (37 °C). The total enzyme activity and specific enzyme activity of the reaction system were calculated using a TOOS extinction coefficient of 18.44 × 10^−3^ μM ^−1^cm^−1^. The abbreviations and optimum pH of various enzymes are shown in Table 1.

**Table 1:** Abbreviations and optimum of various reagent components.

Reagents	Abbreviation	Optimum pH
Guanase	GD	6.8–7.7
Xanthine Oxidase	XOD	7.5–8.0
Uricase	UAO	8.5
Superoxide dismutase	SOD	9.0
Catalase	CTA	7.0
Peroxidase	POD	6.0–7.0
4-Aminoantipyrine	4-AA	–
N-ethyl-N-(2-hydroxy-3-sulfopropyl) -3-methylaniline	TOOS	–

### 2.4 Substrate selection

It is well known that GD has many substrates, such as guanine, 8-aza-guanine, 1-methyl-guanine and 6-thio-guanine, etc [21]. The selection of substrate is vital for enzymatic reaction. The substrate with the lowest Km value is the optimal substrate for enzyme. The Km value of substrates are presented in Table 2. According to the Km value, guanine was selected as the best substrate in this experiment.

**Table 2:** The selection of substrate.

Substrates	Km
Guanine	1.05 × 10^−5^
8-aza-guanine	1.02 × 10^−4^
1-methyl-guanine	2.7 × 10^−3^
6-thio-guanine	8 × 10^−4^

### 2.5 Method validation

Validation of the assay was performed based on common scientific indications [22,23], and the predicaments of the ISO 15189 quality standards for clinical laboratories. To verify the accuracy and reproducibility of the developed method, parameters evaluated were reagent blank (RB), sensitivity, accuracy, inter-batch difference, intra-batch difference, linear range.

To verify the stability of the reagent, reagent blank test was conducted using deionized water as a sample for thirty consecutive days, removing the influence of background signals or impurities caused by reagents to improve the accuracy and reliability of analytical results. The accuracy of method is usually verified by conducting recovery test when establishing a method. By adding a quantitative standard substance to the blank sample matrix without the tested substance, the recovery rate is calculated based on the ratio of the obtained result to the theoretical value according to sample processing analysis, reflecting the accuracy of the test results. The recovery of the method were determined by analyzing six samples at three different levels (3.0, 4.5, 6.0 U/L). Accuracy should be within 85–115 %. Precision (coefficient of variation, CV) are assayed by analyzing six sets of replicates of samples. Samples at low and high levels (1.5,15 U/L)were evaluated in three batches to examine the intra- and inter-day precision of the method. For intra-day analysis six replicates at each level of QC concentration within the batch were examined, whereas for the inter-day analysis, 18 replicates from all the batches were examined. The precision should not exceed 15 % of CV. Linear range refers to the relationship between the detection result and the sample concentration within a certain concentration range, reflecting the sensitivity of the reagent. Usually, a series of concentration samples using dilution method were tested, drawing a calibration curve. Calibration curves were constructed by plotting mearured activity (y) of versus its nominal concentration (x) using a 1/x2 weighting factor. An acceptable determination coefficient (r > 0.995) was obtained.

### 2.6 Stability of GD kits

A total of 3 stabilizers were identified from the literature. These protective agents include gentamicin sulfate, bovine serum albumin (BSA), mannitol. Using the above reaction system, enzyme activity was measured at different concentrations of stabilizers to determine the optimal concentration.These stabilizers concentrations were set as follows and divided into five groups: (i)control groups, without additives; (ii) gentamicin sulfate (0.001 g/L), BSA (0.1 g/L), mannitol (10 g/L); (iii) gentamicin sulfate (0.1 g/L), BSA (1 g/L), mannitol (20 g/L); (iv)gentamicin sulfate (0.2 g/L), BSA (2.0 g/L), mannitol (30 g/L); (v) gentamicin sulfate (0.5 g/L), BSA (3.0 g/L), mannitol (50 g/L). Each experiment was repeated three times. Activity was measured as described above at 0 and 22nd day. Enzyme activity in the absence of any additives was used as the control (100 %). Long-term stability was studied on analysis with a specific concentration of composite stabilizer for 12 months.

### 2.7 Statistical analysis

Data acquisition, data processing and graphic presentation were performed with Microsoft 2010 (Microsoft, Bellevue, USA) and GraphPad Prism 6.01 (GraphPad Software, USA), respectively.

## 3 Results and discussion

### 3.1 Optimization of reaction system and substrate specificity

GD is well established as an indicator of liver disease, and it provides a simple and reliable method for monitoring liver function during early acute liver injury such as viral hepatitis, post transfusion hepatitis and toxic hepatitis [24,25]. It have been advocated a number of methods for the estimation of GD activity. The most commonly used method is enzyme-coupling assays described include using xanthine oxidase and peroxidase with a final oxidation of a chromogen. Traditional enzyme coupling reactions rely solely on the monomolecular H_2_O_2_ generated from oxidation of xanthine [26]. In this study, a two-way enzyme coupling reaction based on xanthine oxidase and uric acid oxidase was established, which produced twice as much H_2_O_2_, improving the sensitivity of this method. We selected that coupling the following four enzymes makes possible quantification of GD: GD (EC 3.5.4.3), xanthine Oxidase (EC 1.1.3.22), uricase (EC 1.7.3.3) and peroxidase (EC 1.11.1.7) (Fig. 1). The enzymes in GD kit were studied for its optimum pH. The reaction with 0.1 mM guanine performed at various pH demonstrated that the GD converted guanine to xanthine most efficiently at pH 6.8–7.7. pH stability test demonstrated that various enzymes retained full activity level at pH 6.5–10.0. The optimal pH of various tool enzymes is shown in Table 1. For this purpose of removing endogenous substances, the solution I used for pretreatment of measurement sample was added with uricase and catalase, which eliminates uric acid and hydrogen peroxide generated due to the oxidation of xanthine, respectively. Catalase included in solution I should be inactivated in the reaction because it degrades hydrogen peroxide generated due to the reaction of xanthine. For this purpose, solution II was added with sodium azide inhibiting catalase activity. The solution I also contained ascorbate oxidase eliminating ascorbic acid existing in the measurement sample that can decolorize the final dye product due to its reducing activity in the reaction. The serial catalytic reaction produces a pigment due to the oxidative coupling between 4-AA and Trinder's reagent (TOOS). We also tested other Trinder's reagent, but their dye formation efficiency was lower than that of TOOS (data not shown). We expected that the initial concentration of GD can be quantified by photometrically measuring the final dye concentration. GD exhibited a high substrate specificity. Kinetic analysis of the reaction with GD demonstrated at 0.1 M guanine and 37 °C, which affinity was significantly higher than that reported previously (Table 2). Km value of GD for guanine was 0.011 mM. The method of GD concentration measurement by using the finally constructed kit is summarized in Fig. 1 (see materials and methods).

### 3.2 Method validation

**Fig. 3:**
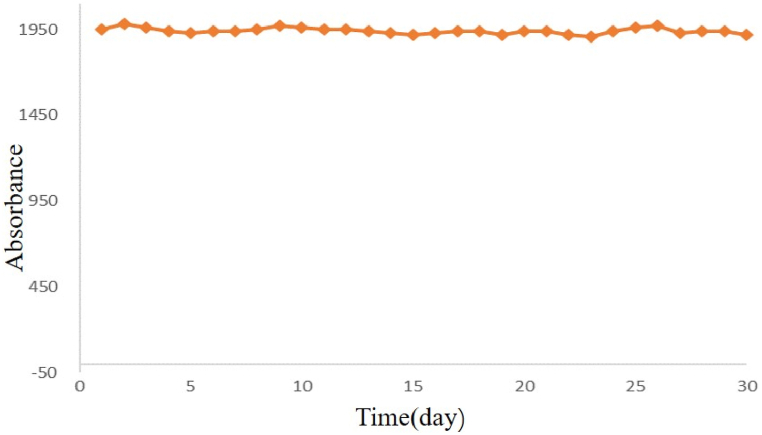
Reagent blank.

**Fig. 4:**
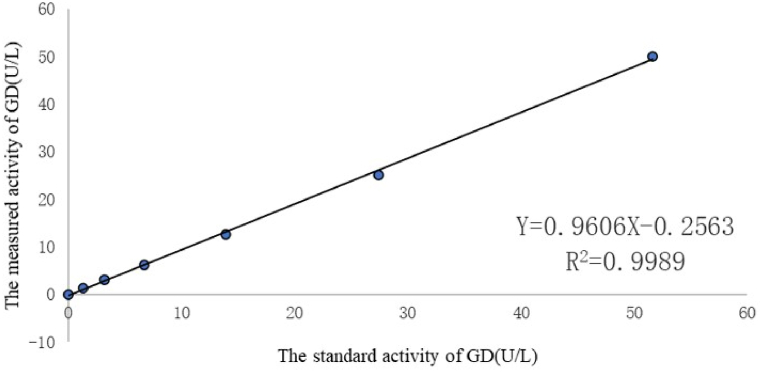
Calibration curve.

As shown in Table 1, Table 2, we explored the performance of this method under optimal conditions. For RB test, The change of reagent blank was ΔΑ = 0.19–0.20 AU (absorbance unit) when stored for four weeks at 2–8 °C (Fig. 3). The sensitivity of this method at 600 nm was ΔΑ = 0.028 AU per min.We tested the linearity with a containing 50 U/L GD, which was stepwise diluted with physiological saline. Fig. 4 shown that the method was linear in the range 0–50 U/L with typical regression equations of y = 0.9606x-0.2563 (R^2^ = 0.99). It is generally believed that a correlation coefficient R^2^ more than 0.99 indicates that the linearity of the reagent kit meets requirement. The recovery test results are displayed in Table 3. The recoveries for three concentrations of GD ranged from 98.2 to 103.3 %, implying that the recoveries were reproducible and concordant across all the concentration ranges in this study. The precision and accuracy were evaluated at the low and high levels. The results were within the required ranges, as shown in Table 4. The CV values for intra- and inter-day precision were 1.62%–3.84 % and 2.69%-6.82 % within the acceptance limits of ≤15 %, respectively. The accuracies for all concentration were within 93.5%–98.5 % in this method.

**Table 3:** Extraction recoveries of GD in hunman serum (n = 6).

Compound	Concentration (U/L)	Recovery (%)	
		Average	RSD
GD	3.0	101.3	0.29
	4.5	103.3	0.33
	6.0	98.2	0.22

**Table 4:** Precision and accuracy and for GD in human serum.

Compound	Concentration (U/L)	Intra-day (n = 6)		Inter-day (n = 18)	
		Accuracy (%)	Precision (CV, %)	Accuracy (%)	Precision (CV, %)
GD	1.5	94.4 ± 9.5	3.84	93.5 ± 9.7	6.82
	15	97.1 ± 10.1	1.62	98.5 ± 5.9	2.69

### 3.3 Stability of GD kits

**Fig. 5:**
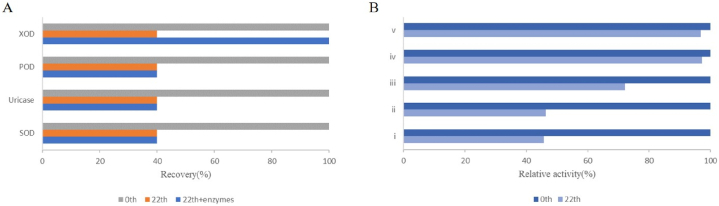
Degradation mechanism study(A) and composite stabilizer concentration determination(B).

For liquid biochemical reagents, enzymes stability take into consideration. Enzymes are beneficial for preservation and transportation, but sometimes leads to loss of enzyme activity, and the addition of a protective agent is needed for protein stabilization. The residual activity of enzymes decreases with the prolongation of storage time, leading to low sensitivity. Therefore, it is necessary to add protective agents to stabilize the protein, protect the protein native structure from denaturation, and reduce the loss of enzyme activity [27]. For the degradation mechanism study, it was found that the GD values recovery to their initials only when the required concentration of XOD was added on the 22nd day (Fig. 5A). These results indicated that the XOD activity is the main factor affecting the stability of the kit. Various compounds, such as sugars, polyols, amino acids, proteins, and polymers have been proven to be effective in minimizing protein denaturation [28]. The stability of a protein results from counteracting enthalpic and entropic contributions. As the close relation between the structure and dynamics of solvent and protein flexibility and stability, the coordination of water molecules with a cosolvent may have a great influence on protein stability [29]. To date, several theories have been proposed to explain the protective mechanisms explaination for the effects of protectants on proteins [30]. Vitrification and water replacement theory are the two main mechanisms. The latter mechanism involves the formation of hydrogen bonds between stabilizers and polar groups of protein molecules, inhibiting the unfolding of proteins. Other stabilization mechanisms contain ligand binding, protectant-protein interactions via amino protons and accumulation of stabilizers around specific amino acid types [31]. A total of 3 stabilizers, including gentamicin sulfate, bovine serum albumin, and mannitol, were used to stabilize enzymes activity in this study.

For the composite stabilizers, different concentration stabilizers were added to deternine thier optimal concentration. The concentrations were set see Materials and method. As shown in Fig. 5B, When no composite stabilizer was added, the GD activity showed a significant downward trend over time. There were no difference between control group and group (i). That is, they had no protective effect on the kit when the content of composite stabilizer were low. In addition, As the concentration of each component in the composite stabilizer subsequent increases, there is no higher change in its stability effect because its solubility in the kit decreases. Similarly, the highest stability effect was observed at group (iv). The concentration of the composite stabilizer is as follows: gentamicin sulfate (0.2 g/L), BSA (2.0 g/L), mannitol (30 g/L).

**Fig. 6:**
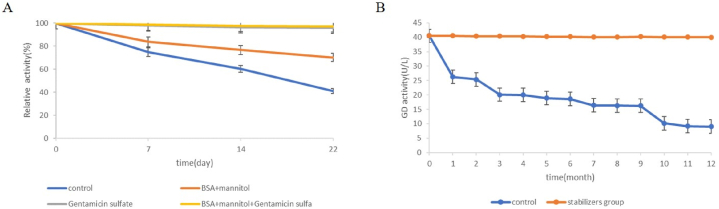
Effects of composite stabilizer on GD activity(A) and long-term stability(B).

After determining the optimal concentration of composite stabilizers, we verify the protective effect of each component of the stabilizer by adding gentamicin, bovine serum albumin, and mannitol, respectively. Research has found that all three components have stable effects, but the combination of the three has a better effect (Fig. 6A). It is that a single stabilizer only has a good stable effect on the target enzyme, which even inhibitory effects on other enzymes. Due to the presence of multiple enzymes in this kit, the addition of gentamicin, bovine serum albumin, or mannitol individually can lead to the stabilizers effects to counteract each other, exhibiting a stabilizing effect on one enzyme and an inhibitory effect on another enzyme in the kit.

In this study, the effects of composite stabilizers in long-term storage were tested. Compared to the control group without additives, the stabilizer group maximally preserved the bioactivity of the enzymes stored for 12 months (Fig. 6B). As described above. enzymes with high stability has great advantages in clinical applications. This is because when GD kit are used for liver function tests, and enzymes, which is highly stable, can help obtain reliable data, which is highly significant in clinical detection of disease.

## 4 Conclusion

In this study, we successfully developed and validated a new method with high sensitivity and strong stability for GD activity quantification based on the double enzyme-couping technique. The developed method was validated as per the FDA guidelines for bioanalytical methods. The design of this reaction system effectively enhance the sensitivity of GD kit. Besides, this proposed method shows high stability as the additions of composite stabilizer.

## Funding statement

This work is supported by Jilin Provincial Department of Education (JJKH20241336KJ).

## Data availability statement

The data presented in this study are available upon request from the corresponding author.

## CRediT authorship contribution statement

**Qiang Sun:** Writing – review & editing, Writing – original draft, Visualization, Validation, Software, Methodology, Funding acquisition. **Haidi Gao:** Supervision, Resources, Project administration. **Yong Liu:** Resources, Methodology, Investigation, Conceptualization. **Liqiang Wang:** Visualization, Supervision, Investigation. **Jing Huang:** Methodology, Investigation, Formal analysis, Conceptualization.

## Declaration of competing interest

The authors declare no conflict of interest.
